# BD-PaveSurface: A multi-weather pavement surface image dataset for crack, pothole, and good pavement condition assessment in Bangladesh

**DOI:** 10.1016/j.dib.2026.113180

**Published:** 2026-08-18

**Authors:** Durjoy Kumar Dutta, Utsho Kundu, Nakib Aman, Al Arian Ahmad, Mohammed Jan-E-Alam Fouad, Nymur Rahaman Antor

**Affiliations:** a Department of Computer Science and Engineering, Pabna University of Science and Technology, Pabna 6600, Bangladesh; b Department of Civil Engineering, Ahsanullah University of Science and Technology, Dhaka, Bangladesh

**Keywords:** Pavement image dataset, Pavement condition assessment, Crack recognition, Pothole recognition, Multi-weather imaging, Computer vision, Road condition monitoring

## Abstract

This data article describes BD-PaveSurface, a pavement surface image dataset developed for image-level assessment of three pavement surface conditions: crack, pothole, and good pavement surface. The images were collected through systematic field surveys from selected segments of National Highways N5 and N6 in Pabna District, which form part of the Pabna-Dhaka highway route in Bangladesh. Smartphone cameras were used to capture pavement surface images under natural outdoor lighting and real traffic-environment conditions, without interrupting normal traffic movement. Data collection was conducted over an extended period across rainy-season, winter, and summer conditions to capture realistic variation in pavement appearance, illumination, surface moisture, shadows, dust, and texture. The collected images underwent manual screening and image-level labeling. Labeling was conducted by a five-member annotation team, with each image independently reviewed by a second team member. Disagreements were resolved through consensus using predefined class criteria and the dominant-condition rule for mixed crack–pothole cases, followed by independent validation by a Civil Engineering faculty member. After removal of unsuitable samples and label verification, the cleaned raw dataset contained 9000 field images, with 3000 images in each pavement condition class. To support machine-learning-based pavement condition assessment, a processed/augmented subset was prepared from the cleaned images. The preprocessing workflow converted images to RGB format and resized them to 224 × 224 pixels. The cleaned images were split into training, validation, and testing subsets before augmentation, and augmentation operations were then applied only to the training subset. The validation and testing subsets contained only standardized original images and were not augmented. The cleaned raw dataset represents 9000 unique field-acquired pavement images. The processed/augmented subset contains 12,000 derivative image files generated from these field-acquired images, comprising standardized 224 × 224 versions of all 9000 cleaned originals and 3000 newly generated training-only augmented images (1000 per class). Accordingly, the dataset contains 21,000 stored image files in total; however, these files do not represent 21,000 distinct field acquisitions or physical pavement locations. Publicly available Bangladesh-focused pavement image datasets containing both crack and pothole samples remain limited. BD-PaveSurface contributes a field-collected, multi-weather pavement image resource from a South Asian road environment and can be reused for automated pavement condition assessment, crack and pothole recognition, transfer learning, data augmentation studies, computer vision benchmarking, and low-cost camera-based road monitoring.

Specifications Table

**Table:** 

Subject	Engineering and Materials Science
Specific subject area	Image-based pavement condition assessment and road surface distress analysis.
Type of Data	Images and tabular metadata.
Data format	Raw and processed/augmented pavement images in standard image formats, together with image-level metadata in CSV format.
Data collection	Pavement surface images were captured using smartphone cameras during field surveys from selected segments of National Highways N5 and N6 in Pabna District, Bangladesh. Images were collected under natural outdoor lighting across rainy-season, winter, and summer conditions. The images were manually reviewed, cleaned, labeled, and organized into three image-level pavement condition classes: Crack, Pothole, and Good.
Data source location	Selected sections of National Highways N5 and N6, Pabna District, Bangladesh. Approximate geographic range: 23.90° to 24.15° N latitude and 89.20° to 89.65° E longitude.
Data accessibility	Repository name: Zenodo Dataset DOI: 10.5281/zenodo.21855576 Direct URL: https://doi.org/10.5281/zenodo.21855576
Related research article	None

## 1 Value of the Data

• The dataset provides a pavement image resource for three road surface conditions: crack, pothole, and good pavement surface, supporting image-level pavement condition assessment and supervised model development.
• It contributes Bangladesh-focused pavement distress imagery from selected sections of National Highways N5 and N6 in Pabna District, where open image datasets for crack and pothole assessment remain limited.
• The inclusion of images captured across rainy-season, winter, and summer conditions increases visual diversity and reflects practical variations in surface moisture, illumination, shadows, dust, pavement color, roughness, and texture.
• The availability of both raw field images and processed/augmented images allows users either to design their own preprocessing workflow or to directly use the prepared dataset for machine-learning experiments.
• The dataset can support researchers, transportation engineers, and computer vision practitioners working on automated pavement condition assessment, crack and pothole recognition, transfer learning, augmentation strategy evaluation, and road infrastructure monitoring.
• The dataset can support the development of lightweight, camera-based road condition assessment models suitable for low-cost and resource-constrained road monitoring applications in Bangladesh and similar South Asian environments.

## 2 Background

Road pavement distress, particularly cracks and potholes, is a visible indicator of surface deterioration and can affect ride quality, traffic safety, vehicle operating cost, and long-term pavement performance. Traditional pavement inspection often depends on manual visual assessment, which is time-consuming and subject to observer variation. Image-based pavement condition assessment has therefore become an important research direction, with early studies showing the use of computer vision for pothole detection in asphalt pavement images [1]. Deep learning methods have also advanced automated crack detection and pavement defect recognition from road surface images [2]. However, crack and pothole recognition remains challenging because pavement images are affected by illumination variation, shadows, low contrast, water, dust, rough texture, and complex surface backgrounds. Smartphone-based road imagery has been used for automated road damage detection and classification using deep neural networks [3]. Convolutional neural network methods have also been applied to pavement crack detection, segmentation, and distress analysis under varying image conditions [4]. Model performance, however, depends strongly on representative datasets that capture field variation across devices, weather conditions, pavement textures, and surface states [5]. Open datasets such as RDD2020 have supported reproducible road damage detection research [6], while recent resources such as PaveDistress have further expanded pavement distress datasets for detection and analysis [7]. Despite these developments, publicly available Bangladesh-focused pavement image datasets containing both pothole and crack samples remain limited. This article addresses that gap by presenting a field-collected, multi-weather pavement image dataset from selected N5 and N6 highway sections in Pabna District, Bangladesh. The dataset contains three image-level classes: crack, pothole, and good pavement surface, and can support automated pavement condition assessment, crack and pothole recognition, transfer learning, computer vision benchmarking, and low-cost camera-based road monitoring. The balanced class distribution, multi-season field coverage, and leakage-controlled dataset preparation provide additional support for reproducible dataset reuse and evaluation.

## 3 Data Description

**Fig. 1:**
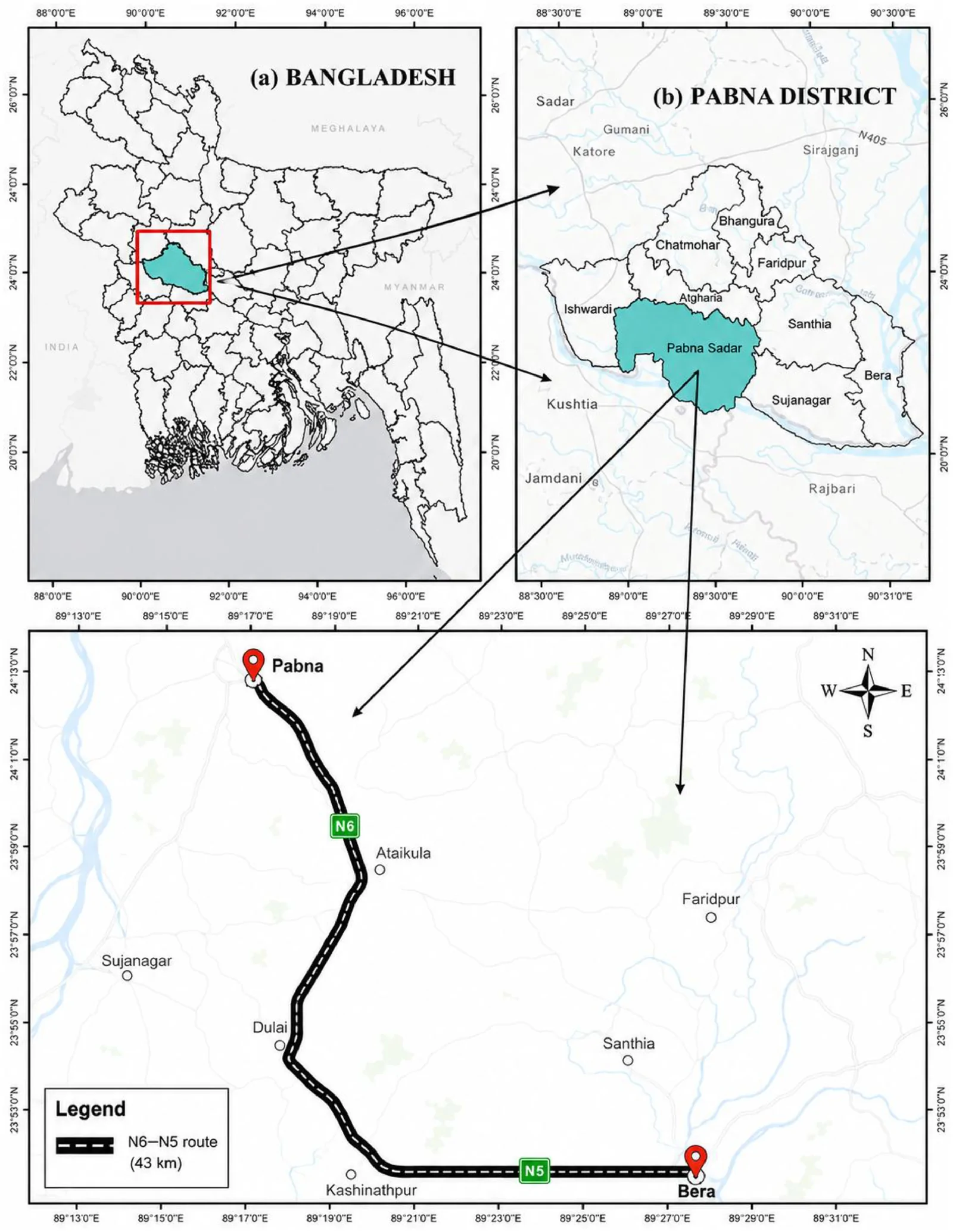
Location of the pavement image collection area along selected N5 and N6 sections in Pabna District, Bangladesh.

The dataset contains pavement surface images prepared for image-level assessment of three pavement surface conditions: Crack, Pothole, and Good pavement surface. The images were collected from selected segments of National Highways N5 and N6 in Pabna District, Bangladesh, within the approximate geographic range of 23.90° to 24.15° N latitude and 89.20° to 89.65° E longitude. The data collection area is shown in Fig. 1. The complete BD-PaveSurface dataset contains 9000 unique field-acquired images and 12,000 derivative processed/augmented files, resulting in 21,000 stored image files in total.

**Fig. 2:**
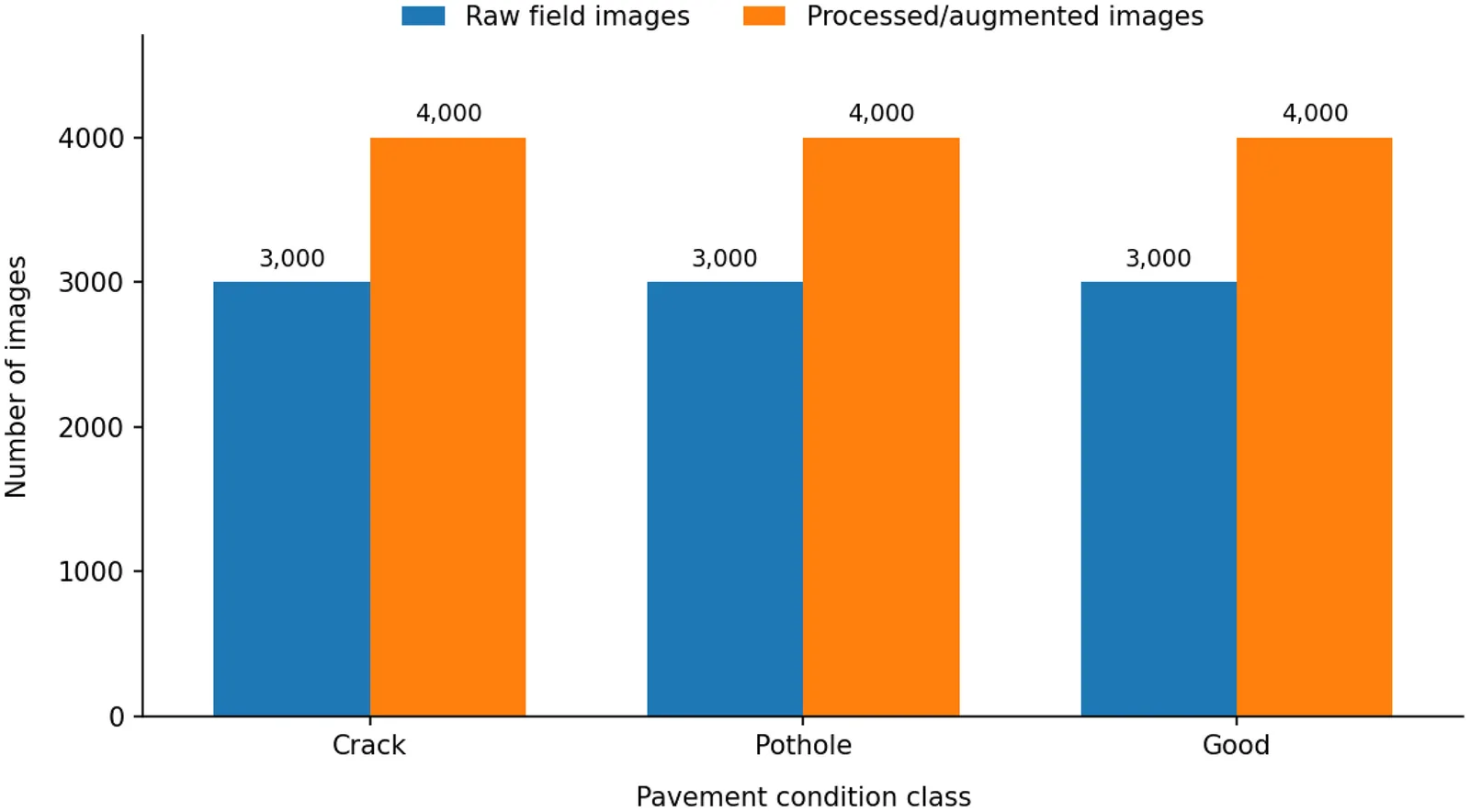
Class-wise distribution of raw and processed/augmented images in the Crack, Pothole, and Good classes.

The dataset is organized into three pavement condition classes: Crack, Pothole, and Good. The raw subset contains 3000 field images per class, resulting in 9000 original images. The processed/augmented subset contains 12,000 images, comprising RGB-standardized 224 × 224 versions of the 9000 cleaned original images and 3000 newly generated training-only augmented images (1000 per class). The class-wise distribution of the dataset is presented in Table 1 and visualized in Fig. 2.

**Table 1:** Class-wise distribution of the pavement surface image dataset.

Dataset component	Crack	Pothole	Good	Total
Unique field-acquired original images	3000	3000	3000	9000
Derivative processed/augmented files	4000	4000	4000	12,000
**Total stored image files**	7000	7000	7000	21,000

**Note:** The 12,000 processed/augmented files are derived from 9000 unique field-acquired images. Thus, 21,000 stored files do not represent 21,000 distinct field acquisitions or physical locations.

To document the seasonal coverage of BD-PaveSurface, the 9000 cleaned original pavement images were categorized according to the corresponding field-survey season. The dataset contains 2575 images from rainy-season surveys, 2300 images from winter surveys, and 4125 images from summer surveys. The detailed class-wise seasonal distribution is summarized in Table 2. An accompanying image-level metadata CSV file is provided for the cleaned original images and includes the image identifier, filename, pavement-condition class, seasonal category, camera-device information, and collection-date information where available.

**Table 2:** Seasonal distribution of the 9000 cleaned original pavement images.

Season	Crack	Pothole	Good	Total
Rainy season	725	1300	550	2575
Winter	850	650	800	2300
Summer	1425	1050	1650	4125
**Total**	3000	3000	3000	9000

**Fig. 3:**
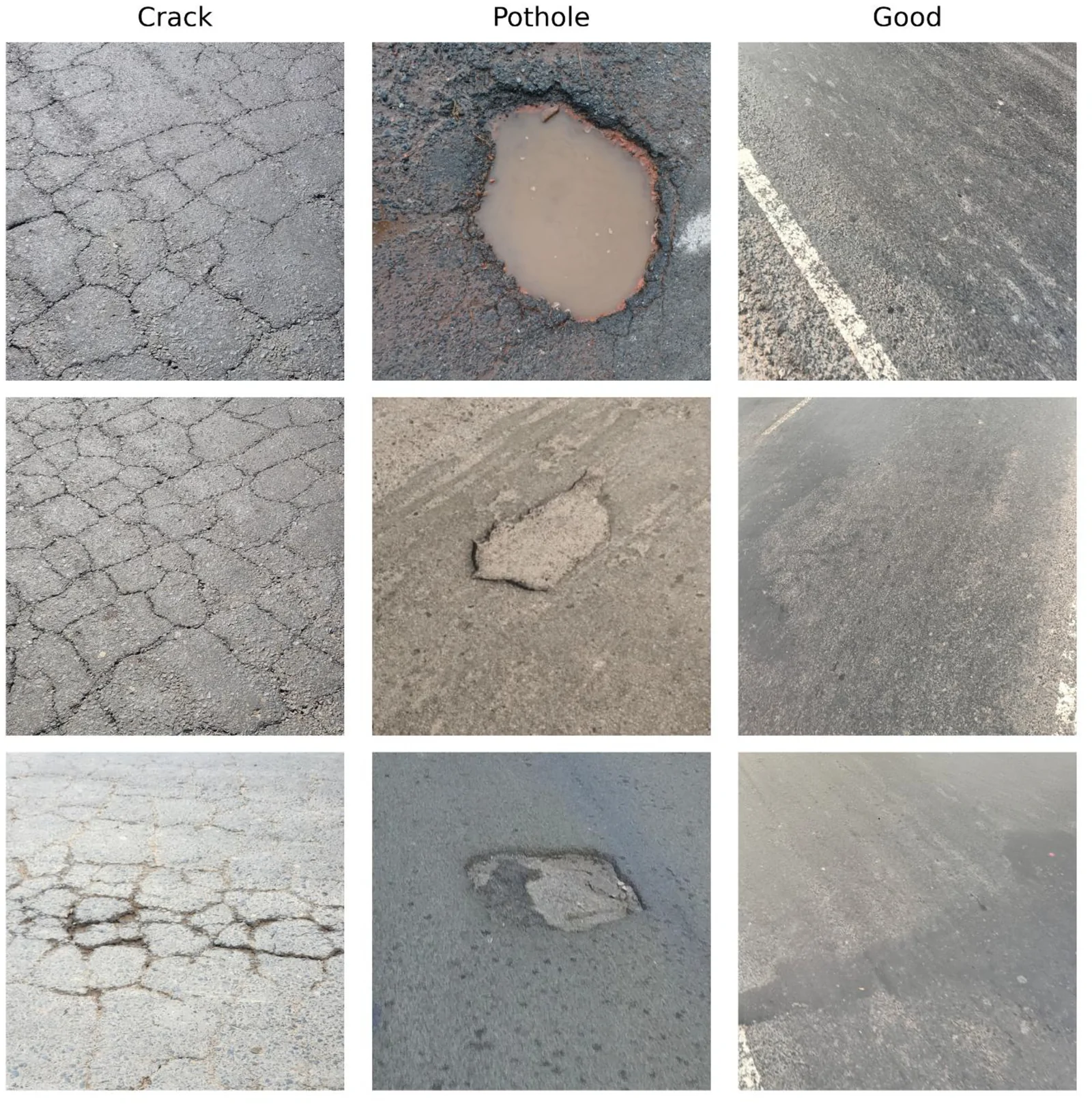
Representative pavement surface images from the Crack, Pothole, and Good classes.

The Crack class contains pavement images with visible surface cracking, including linear, longitudinal, transverse, and interconnected crack patterns. The Pothole class contains pavement images showing localized surface failure, including dry potholes and water-affected potholes. The Good class contains pavement images without visually dominant cracks or potholes and represents intact or serviceable pavement surface conditions. Images in the Good class may still contain normal pavement texture, dust, shadows, lane markings, surface color variation, or minor roughness. Representative samples from the three classes are shown in Fig. 3.

All images are labeled at the image level according to the dominant pavement condition visible in the frame. The dataset does not include pixel-level segmentation masks, bounding boxes, crack-width measurements, pothole-depth measurements, or distress severity scores. Therefore, the dataset is mainly suitable for image-level pavement condition assessment, crack and pothole recognition, transfer learning, data augmentation studies, and road surface classification tasks.

The processed/augmented subset is arranged into training, validation, and testing folders using a 70:15:15 split of the 9000 cleaned original images. The original images were assigned to the three subsets before augmentation using the leakage-controlled splitting procedure. This resulted in 2100 original training images, 450 original validation images, and 450 original testing images per class. Data augmentation was subsequently applied only to the training subset, generating 1000 additional images per class. Consequently, each class contains 3100 training images, comprising 2100 standardized original images and 1000 augmented images, together with 450 validation images and 450 testing images. The validation and testing subsets contain only standardized original images and were not augmented. The distribution of the processed/augmented subset is shown in Table 3.

**Table 3:** Distribution of the processed/augmented subset.

Class	Training	Validation	Testing	Total
Crack	3100	450	450	4000
Pothole	3100	450	450	4000
Good	3100	450	450	4000
**Total**	9300	1350	1350	12,000

**Fig. 4:**
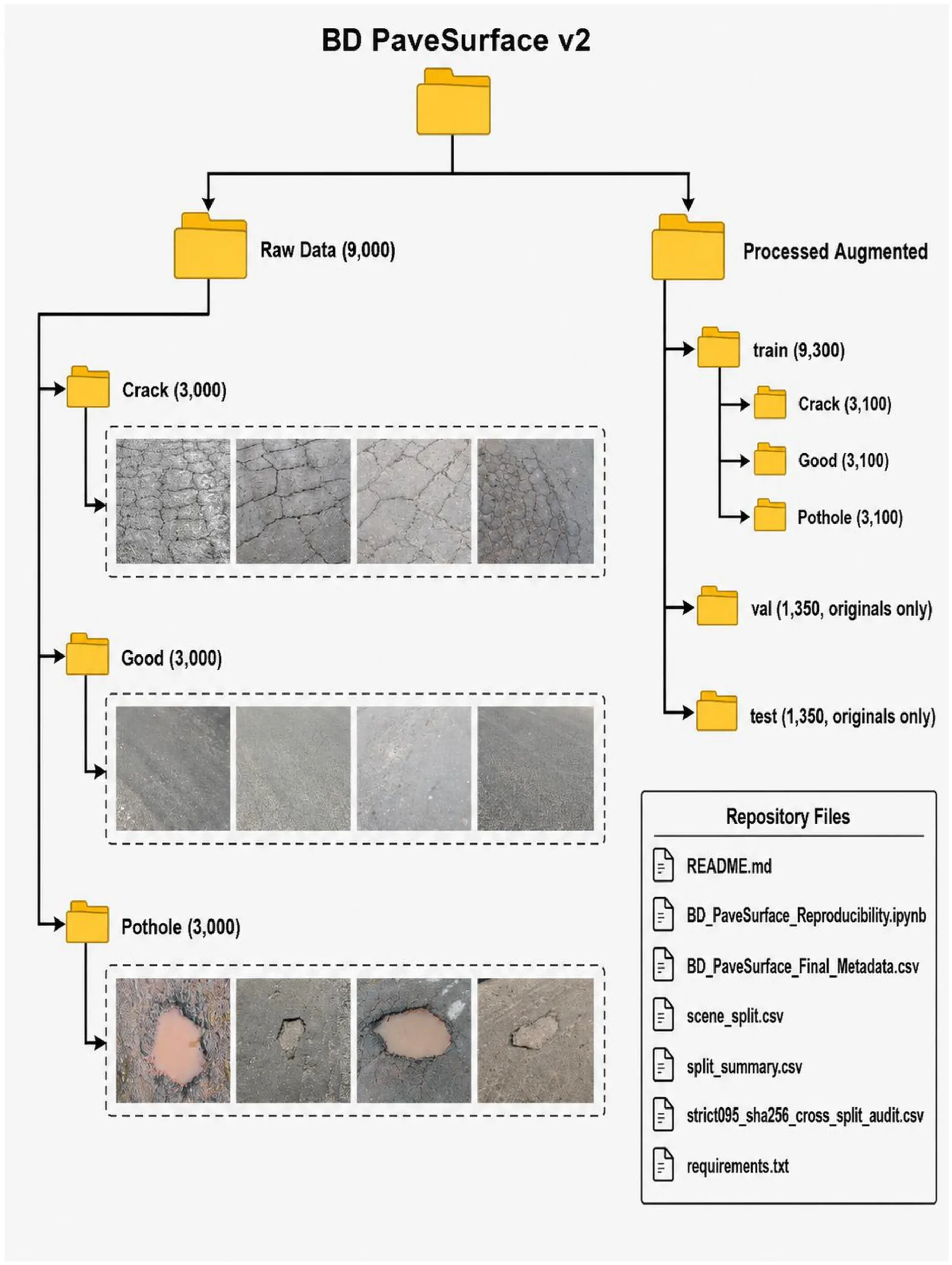
Folder structure of the raw and processed/augmented pavement image dataset.

The dataset is organized in class-wise folders to support direct use in common computer vision workflows. The raw subset contains original field images grouped into Crack, Good, and Pothole folders. The processed/augmented subset contains resized and augmented images divided into train, val, and test folders, and each split contains the same three class folders: Crack, Good, and Pothole. The dataset folder structure is shown in Fig. 4.

## 4 Experimental Design, Materials and Methods

**Fig. 5:**
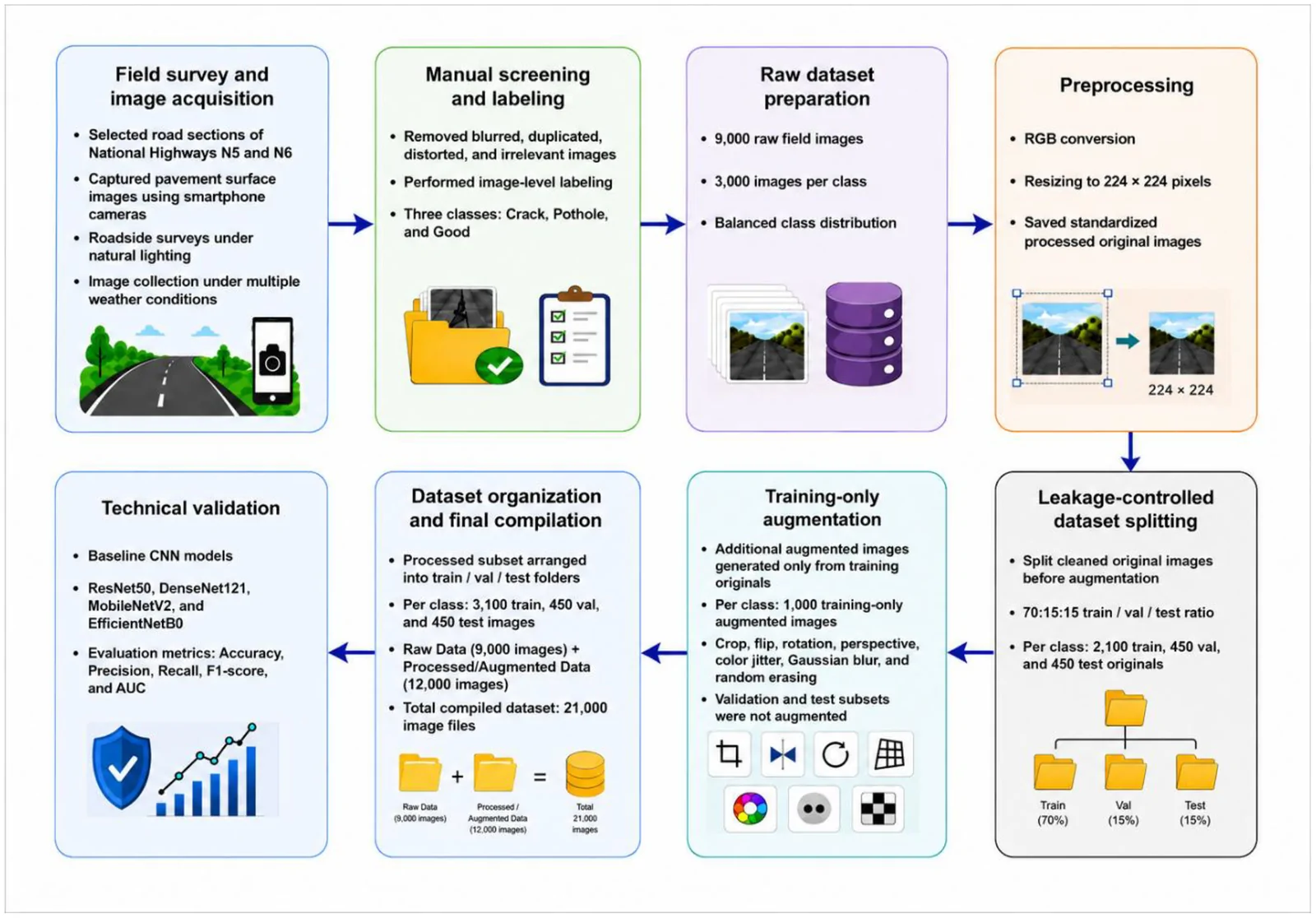
Workflow of pavement image collection, dataset preparation, organization, and technical validation.

The dataset was developed through a sequential workflow consisting of field survey planning, pavement image acquisition, manual screening, image-level labeling, preprocessing, dataset splitting and augmentation, dataset organization, final compilation, and technical validation. The overall process of pavement image collection, cleaning, preprocessing, leakage-controlled dataset splitting, training-only augmentation, dataset organization, and validation is shown in Fig. 5. The workflow was designed to prepare a structured pavement surface image dataset for three pavement surface condition classes: Crack, Pothole, and Good.

### 4.1 Field survey design and image acquisition

A preliminary reconnaissance survey was conducted before the main field image collection to identify suitable road sections, observe pavement surface conditions, and plan the data acquisition process systematically. Pavement surface images were collected from selected segments of National Highways N5 and N6 in Pabna District, Bangladesh. The survey area was located within the approximate geographic range of 23.90° to 24.15° N latitude and 89.20° to 89.65° E longitude, as shown in Fig. 1. The selected road sections contained pavement surfaces representing cracked pavement, pothole-affected pavement, and good pavement surface conditions.

Images were acquired during roadside field surveys using multiple smartphone cameras, including the Redmi K50i, iPhone 15 Plus, and vivo X200 Pro. The photographs were captured under natural outdoor lighting and real traffic-environment conditions. No artificial illumination, laboratory setup, or fixed imaging platform was used. During image acquisition, the camera was directed toward the pavement surface so that the road condition occupied the main part of the image frame. To ensure systematic data collection, images were captured from selected road sections following a consistent field procedure, including pavement-focused framing, class-wise visual observation, and repeated checking of image clarity during collection. Standard field safety practices were followed during the surveys, and images were captured without interrupting normal traffic movement.

The field surveys were conducted over an extended period covering rainy-season, winter, and summer conditions. This seasonal coverage introduced natural variation in pavement appearance, including differences in illumination, shadows, dust, surface moisture, pavement color, roughness, and texture. The quantitative seasonal distribution of the cleaned original images is reported in Table 2.

### 4.2 Class definition and labeling protocol

The collected images were labeled into three image-level pavement condition classes: Crack, Pothole, and Good. Each label was assigned according to the dominant pavement condition visible in the image.

The Crack class includes pavement images with visible surface cracking, such as linear cracks, longitudinal cracks, transverse cracks, and interconnected crack patterns. The Pothole class includes images showing localized pavement surface failure, including dry potholes and water-affected potholes. The Good class includes pavement surface images without visually dominant cracks or potholes and represents intact or serviceable pavement surface conditions. Images in this class may still include normal pavement texture, dust, shadows, lane markings, surface color variation, or minor roughness.

All labels were assigned at the image level. The dataset does not include pixel-level segmentation masks, bounding-box annotations, crack-width measurements, pothole-depth measurements, or distress severity scores. If more than one pavement condition appeared in the same image, the final label was assigned based on the dominant visible road condition. For example, an image containing a pothole with surrounding cracking was assigned to the Pothole class when the pothole was the primary visible distress.

The image-level labeling process involved five individuals, including three participants with Computer Science and Engineering backgrounds and two with Civil Engineering backgrounds. Each image received an initial label from one member of the annotation team and was subsequently independently reviewed by another member as a quality-control step. When disagreement occurred, the image was discussed by the reviewers and the final label was assigned through consensus according to the predefined class criteria and dominant-condition rule. In addition to the five-member annotation team, a Civil Engineering faculty member independently performed expert validation of the final labels. A formal inter-annotator agreement statistic was not calculated because the workflow followed sequential labeling, review, consensus-based disagreement resolution, and subsequent expert validation rather than fully independent annotation of the complete dataset by multiple annotators.

### 4.3 Manual screening and dataset cleaning

After field acquisition, all collected images were manually reviewed before final dataset preparation. Images were excluded if they were severely blurred, distorted, duplicated, incorrectly framed, unrelated to pavement surface condition, or unsuitable for image-level pavement condition assessment. Images with unclear class identity were also removed during manual screening.

After this cleaning process, the raw dataset contained 9000 field images, with 3000 images in each class. The cleaned raw images were organized into three class-wise folders: Crack, Good, and Pothole. These raw images preserve the original field-collected pavement surface appearance and form the primary observational component of the dataset.

### 4.4 Preprocessing workflow

The cleaned raw images were standardized before generating the processed/augmented subset. The preprocessing workflow was implemented in Python using standard image-processing and deep-learning libraries. Each image was converted to RGB format to ensure consistent channel representation and resized to 224 × 224 pixels to provide a uniform input dimension for image-classification models. The preprocessed images were saved in JPEG format and used as the basis for the processed/augmented subset.

### 4.5 Dataset splitting and augmentation

To minimize scene-level leakage, the 9000 cleaned original images were grouped within each class using L2-normalized ImageNet-pretrained ResNet50 features and cosine similarity with a conservative threshold of 0.95. Visually similar images assigned to the same scene group were kept entirely within a single training, validation, or testing subset. As an additional quality-control step, the largest automatically formed similarity groups were visually inspected through contact sheets to verify that unrelated pavement scenes had not been incorrectly grouped. Cross-split separation was further assessed using a nearest-neighbor similarity audit, including targeted visual inspection of 108 of the strongest cross-split image pairs; all reviewed pairs represented different physical pavement scenes, with no same-scene leakage identified among the inspected pairs. In addition, an SHA-256 file-hash audit was performed to identify exact duplicate files across the training, validation, and testing subsets. No exact cross-split duplicate files were identified by the SHA-256 audit.

Following scene-level grouping, the 9000 cleaned original images were assigned to class-balanced training, validation, and testing subsets using a 70:15:15 ratio. For each class, 2100 original images were assigned to training, 450 to validation, and 450 to testing. Data augmentation was then applied exclusively to the training subset, generating 1000 additional images per class. Consequently, each class contains 3100 training images, comprising 2100 standardized original images and 1000 augmented images, together with 450 validation images and 450 testing images. The validation and testing subsets contain only standardized original images and were not augmented. Overall, the processed/augmented subset contains 9300 training images, 1350 validation images, and 1350 testing images, for a total of 12,000 image files.

The augmentation workflow was implemented using torchvision.transforms and included random resized cropping, horizontal flipping, small-angle rotation, mild perspective transformation, color jittering, Gaussian blurring, and random erasing. These operations introduced controlled variation in image framing, orientation, viewpoint, illumination, color tone, sharpness, and local occlusion while preserving the original image-level class labels. The main preprocessing and augmentation operations used to prepare the processed/augmented subset are summarized in Table 4.

**Table 4:** Preprocessing and augmentation operations used for the processed/augmented subset.

Operation	Setting used	Role in data preparation
RGB conversion	RGB color mode	Standardized image channels
Resize	224 × 224 pixels	Standardized image dimensions
Center crop	224 × 224 pixels	Prepared non-augmented processed images
Random resized crop	224 × 224 pixels; scale 0.75 to 1.00; ratio 0.90 to 1.10	Added scale and framing variation
Horizontal flip	Probability 0.50	Added left-right orientation variation
Small-angle rotation	Maximum 12°; applied with probability 0.70	Added camera-angle variation
Perspective transformation	Distortion scale 0.15; applied with probability 0.25	Added mild viewpoint variation
Color jitter	Brightness 0.20; contrast 0.20; saturation 0.10; hue 0.02	Added lighting and color variation
Gaussian blur	Kernel size 3; sigma 0.1 to 1.0; applied with probability 0.20	Added sharpness variation
Random erasing	Probability 0.25; scale 0.01 to 0.05; ratio 0.3 to 3.3	Added small local occlusion variation
JPEG saving	Quality 95; subsampling 0	Saved processed images in high-quality image format

### 4.6 Dataset organization and final compilation

After preprocessing, leakage-controlled splitting, and training-only augmentation, the processed/augmented subset was organized into train, val, and test folders. Each split contains the same three class folders: Crack, Good, and Pothole. For each class, the training folder contains 3100 images, comprising 2100 standardized original images and 1000 newly augmented images, whereas the validation and testing folders each contain 450 standardized original images only. Accordingly, the processed/augmented subset contains 9300 training images, 1350 validation images, and 1350 testing images, for a total of 12,000 image files. No augmented images are included in the validation or testing subsets. The resulting folder organization is shown in Fig. 4.

After manual screening, preprocessing, splitting, augmentation, and dataset organization, the final dataset was compiled into two main components: the raw field image subset and the processed/augmented subset. The raw subset contains 9000 original field images, with 3000 images in each class. The processed/augmented subset contains 12,000 images, comprising 9000 standardized versions of the cleaned original images and 3000 newly generated training-only augmented images (1000 per class). Together, the final dataset contains 21,000 image files with equal class-wise representation. The raw subset preserves the original field-collected pavement images, while the processed/augmented subset provides standardized images suitable for direct use in supervised image-classification workflows.

### 4.7 Software and computational environment

The preprocessing, augmentation, dataset organization, and technical validation procedures were implemented in Python on a Windows workstation. Image handling and transformation were performed using PIL and torchvision.transforms, while the processed/augmented subset was loaded for validation using the folder-based torchvision.datasets.ImageFolder interface in PyTorch. This loading approach matched the dataset structure because the images were organized into class-wise folders for Crack, Good, and Pothole.

For validation, all images were resized to 224 × 224 pixels, converted to tensor format, and normalized using ImageNet-compatible preprocessing to match the input requirements of the pretrained CNN models. Baseline validation was performed using ResNet50, DenseNet121, MobileNetV2, and EfficientNetB0. All validation experiments were conducted on a workstation equipped with an NVIDIA GeForce RTX 3050 Laptop GPU with 4 GB VRAM, 32 GB RAM, and an AMD Ryzen 7 5800H processor operating at 3.20 GHz. CUDA-enabled GPU acceleration was used during training and inference to improve computational efficiency.

### 4.8 Technical validation workflow

Technical validation was performed to verify the organization, class balance, loadability, leakage-controlled splitting, and machine-learning usability of the processed/augmented subset. The dataset was first loaded using the folder-based torchvision.datasets.ImageFolder interface, which confirmed automatic detection of the three class folders: Crack, Good, and Pothole. The split distribution was also checked, confirming 9300 training images, 1350 validation images, and 1350 testing images, with equal representation of the three pavement condition classes. All 3000 newly augmented images were restricted to the training subset, while the validation and testing subsets contained only standardized original images and were not augmented.

Baseline validation was conducted using four ImageNet-pretrained convolutional neural network architectures: ResNet50, DenseNet121, MobileNetV2, and EfficientNetB0. For each model, the final classification layer was replaced for the three-class pavement condition task. The models were trained using the processed/augmented training subset with the Adam optimizer, cross-entropy loss, a learning rate of 0.0001, weight decay of 0.0001, batch size of 16, and a maximum of 30 epochs. The best checkpoint for each model was selected based on the minimum validation loss and evaluated on the untouched testing subset. Model performance was evaluated using accuracy, loss, weighted precision, weighted recall, weighted F1-score, multi-class AUC, per-class precision, recall, and F1-score, together with confusion matrices.

### 4.9 Baseline validation results

**Fig. 6:**
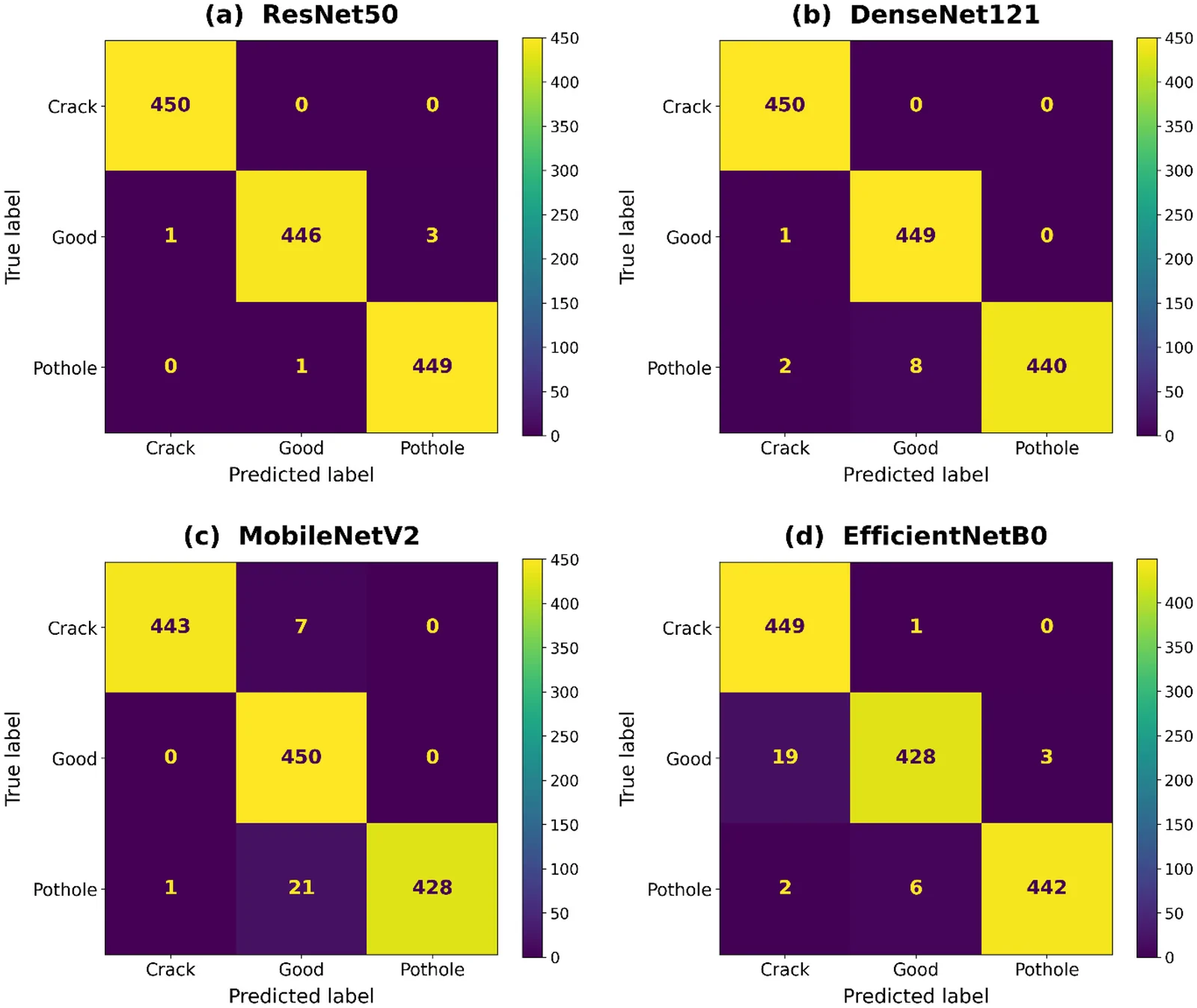
Confusion matrices for baseline classification on the untouched testing subset: (a) ResNet50, (b) DenseNet121, (c) MobileNetV2, and (d) EfficientNetB0.

The baseline classification performance on the untouched testing subset is presented in Table 5. Per-class precision, recall, and F1-score are summarized in Table 6, while the corresponding confusion matrices are presented in Fig. 6. These results provide reference values for future users and demonstrate that the dataset can be used in standard supervised image-classification workflows.

**Table 5:** Baseline classification performance on the untouched testing subset.

Model	Accuracy (%)	Loss	Precision	Recall	F1-score	AUC
ResNet50	99.63	0.027121	0.996303	0.996296	0.996294	0.999956
DenseNet121	99.19	0.032305	0.991957	0.991852	0.991840	0.999965
MobileNetV2	97.85	0.075575	0.979723	0.978519	0.978607	0.999660
EfficientNetB0	97.70	0.064725	0.977495	0.977037	0.977009	0.999290

**Table 6:** Per-class classification performance on the untouched testing subset.

Model	Class	Precision	Recall	F1-score
ResNet50	Crack	0.997783	1.000000	0.998890
	Good	0.997763	0.991111	0.994426
	Pothole	0.993363	0.997778	0.995565
DenseNet121	Crack	0.993377	1.000000	0.996678
	Good	0.982495	0.997778	0.990077
	Pothole	1.000000	0.977778	0.988764
MobileNetV2	Crack	0.997748	0.984444	0.991051
	Good	0.941423	1.000000	0.969828
	Pothole	1.000000	0.951111	0.974943
EfficientNetB0	Crack	0.955319	0.997778	0.976087
	Good	0.983908	0.951111	0.967232
	Pothole	0.993258	0.982222	0.987709

All four pretrained models achieved strong classification performance on the untouched testing subset. ResNet50 achieved the highest accuracy of 99.63%, followed by DenseNet121 with 99.19%, MobileNetV2 with 97.85%, and EfficientNetB0 with 97.70%. The class-wise results showed consistently strong recognition across the Crack, Good, and Pothole classes, with only a limited number of cross-class prediction errors. These findings provide baseline evidence of dataset usability and are not intended to establish model superiority or cross-domain generalization.

Despite the strong baseline performance, dataset-specific visual cues such as pavement background and texture, camera characteristics, moisture and illumination conditions, and image framing may contribute to classification performance in addition to pavement distress features. The similarity-controlled scene-level splitting and leakage-control checks reduce the risk of direct scene overlap; however, broader generalization should be assessed using independently collected road sections, devices, seasons, and environmental conditions. Therefore, the high benchmark accuracies should be interpreted cautiously, and future work should include cross-dataset evaluation to assess the generalizability of the models beyond BD-PaveSurface.

## Limitations

The dataset has several data-related limitations that should be considered when reusing it. It contains three pavement surface categories: Crack, Pothole, and Good, and does not separately represent other distress types such as rutting, raveling, bleeding, patching, edge failure, depression, or general surface wear. The images were collected from selected sections of National Highways N5 and N6 in Pabna District, Bangladesh; therefore, the dataset reflects the pavement materials, traffic exposure, maintenance conditions, and environmental characteristics of this specific road context. Image-level metadata are provided for the cleaned original images, including image identifier, filename, pavement-condition class, seasonal category, camera-device information, and collection-date information. However, exact per-image GPS coordinates, road-section identifiers, standardized instantaneous weather labels, quantitative illumination measurements, surface-wetness measurements, and independently verified capture dates are not available for all images. The annotations are limited to image-level dominant-condition labels and do not include bounding boxes, segmentation masks, or quantitative distress-severity measurements. The processed/augmented subset was also prepared using a fixed workflow, including RGB conversion, resizing to 224 × 224 pixels, and selected augmentation operations. Future extensions may improve reuse by adding more distress categories, expanding image collection to additional road networks, and incorporating more detailed geospatial, environmental, and distress-severity annotations.

## Ethics Statement

This study does not involve human participants, animal experiments, or data collected from social media platforms. The dataset consists of pavement surface images collected during roadside field surveys from selected sections of National Highways N5 and N6 in Pabna District, Bangladesh. Image acquisition was conducted only for documenting road surface conditions and was performed without interacting with road users or interrupting normal traffic movement. During data curation, all images were manually reviewed to ensure that the final dataset focused on pavement surface conditions. Images containing identifiable elements, irrelevant scenes, unsuitable framing, or unnecessary non-road visual content were removed from the final dataset. The authors have read and followed the ethical requirements for publication in *Data in Brief*.

## CRediT Author Statement

**Durjoy Kumar Dutta:** Conceptualization, Methodology, Investigation, Data curation, Software, Formal analysis, Validation, Visualization, Writing (original draft), Writing (review and editing). **Utsho Kundu:** Methodology, Investigation, Data curation, Validation, Visualization. **Nakib Aman:** Supervision, Methodology, Validation, Project administration, Investigation. **Al Arian Ahmad:** Investigation, Data curation, Validation, Visualization. **Mohammed Jan-E-Alam Fouad:** Investigation, Data curation, Validation. **Nymur Rahaman Antor:** Investigation, Data curation, Validation.

## Data Availability

BD-PaveSurface is openly available through Zenodo under the repository title “BD-PaveSurface: A Multi-Weather Pavement Surface Image Dataset for Crack, Pothole, and Good Pavement Condition Assessment in Bangladesh.” The dataset is accessible at https://doi.org/10.5281/zenodo.21855576 and is distributed under the Creative Commons Attribution 4.0 International (CC BY 4.0) license.
